# Structural basis for cytokinin production by LOG from *Corynebacterium glutamicum*

**DOI:** 10.1038/srep31390

**Published:** 2016-08-10

**Authors:** Hogyun Seo, Sangwoo Kim, Hye-Young Sagong, Hyeoncheol Francis Son, Kyeong Sik Jin, Il-Kwon Kim, Kyung-Jin Kim

**Affiliations:** 1 School of Life Sciences, KNU Creative BioResearch Group, Kyungpook National University, Daegu 702-701, Republic of Korea; 2 School of Nano-Bioscience and Chemical Engineering, Ulsan National Institute of Science and Technology (UNIST), Ulsan, 689-798, Republic of Korea; 3 Pohang Accelerator Laboratory, Pohang University of Science and Technology, Jigok-ro 80, Pohang, Kyungbuk 790-784, Korea; 4 Biopoecess Research Depart. R&D Center, DAESANG Corp., Icheon-si, Gyeonggi-do, 467-810, Republic of Korea

## Abstract

“Lonely guy” (LOG) has been identified as a cytokinin-producing enzyme in plants and plant-interacting fungi. The gene product of *Cg2612* from the soil-dwelling bacterium *Corynebacterium glutamicum* was annotated as an LDC. However, the facts that *C. glutamicum* lacks an LDC and *Cg*2612 has high amino acid similarity with LOG proteins suggest that *Cg*2612 is possibly an LOG protein. To investigate the function of *Cg*2612, we determined its crystal structure at a resolution of 2.3 Å. *Cg*2612 functions as a dimer and shows an overall structure similar to other known LOGs, such as LOGs from *Arabidopsis thaliana* (*At*LOG), *Claviceps purpurea* (*Cp*LOG), and *Mycobacterium marinum* (*Mm*LOG). *Cg*2612 also contains a “PGG_X_GT_XX_E” motif that contributes to the formation of an active site similar to other LOGs. Moreover, biochemical studies on *Cg*2612 revealed that the protein has phosphoribohydrolase activity but not LDC activity. Based on these structural and biochemical studies, we propose that *Cg*2612 is not an LDC family enzyme, but instead belongs to the LOG family. In addition, the prenyl-binding site of *Cg*2612 (*Cg*LOG) comprised residues identical to those seen in *At*LOG and *Cp*LOG, albeit dissimilar to those in *Mm*LOG. The work provides structural and functional implications for LOG-like proteins from other microorganisms.

The term cytokinin originated from the cell division-promoting functions of these compounds^1^. Cytokinin phytohormones are usually N^6^-modified adenines such as N^6^-(δ^2^-isopentenyl)adenine (iP) and trans-zeatin (tZ), and they play significant roles in controlling growth and development of plants^2^,^3^. They can be conjugated with sugar moieties such as nucleotides, nucleosides, and glucosides, but these conjugated forms are biologically less active or inactive for plant cytokinin receptors^2^. The cytokinin biosynthetic pathway begins with dimethylallyl pyrophosphate (DMAPP), possibly originating from the mevalonate or methylerythritol phosphate pathway, being prenylated by isopentenyltransferase (IPT) (Fig. 1a). Adenylate-IPT can add DMAPP to ATP/ADP or adenylate, whereas tRNA-IPT modifies the N^6^-atom of adenine moiety on position 37 of tRNA^4^. The isopentenylated products can be converted to the typical metabolite N-(δ-isopentenyl)adenosine 5′-monophosphate (iPRMP) by dephosphorylation or degradation of tRNA^5^. The nucleotide iPRMP might be dephosphorylated by nucleotidase and then deribosylated by nucleosidase to produce an active nucleobase^2^. In 2007, a one-step cytokinin activation pathway was first discovered and the novel cytokinin-activating enzyme called lonely guy (LOG) emerged^6^. LOG produces active cytokinins via dephosphoribosylation, directly hydrolyzing the bond between N^6^-substituted bases and ribose 5′-monophosphates in cytokinin precursors such as iPRMP or trans-zeatin riboside 5′-monophosphate (tZRMP).

For many years, before the discovery of their cytokinin-producing activity, LOGs were known as possible lysine decarboxylases (LDCs) according to the Pfam database, without experimental evidence^7^. Recently, enzymes from several organisms, such as *Oryza sativa*, *Arabidopsis thaliana*, *Claviceps purpurea*, and *Mycobacterium tuberculosis* have been identified as LOGs by biochemical and functional studies^5^,^6^,^8^,^9^. According to morphological and metabolic analyses, the LOG-mediated one-step pathway is suggested as the major cytokinin production pathway and is pivotal for normal growth and development in *Arabidopsis*^8^. Despite a lack of evidence for the phosphoribohydrolase catalytic mechanism, the homodimeric disposition and the active site with highly conserved “PGG_X_GT_XX_E” motif were elucidated by structural studies on LOG proteins^10^,^11^. However, many LOG-like proteins, especially from bacteria, have remained *terra incognita*.

The soil-dwelling bacterium *Corynebacterium glutamicum* has been intensively studied for industrial applications due to its high production of amino acids, nucleotides, and vitamins^12^. Among these products, _L_-lysine has most actively drawn attention in industry^13^. Interestingly, *C. glutamicum* ATCC 13032 contains a gene product of *Cg2612* that is annotated as a possible LDC (pfam03641) and a nucleotide-binding protein. LDCs are known as pyridoxal 5′-phosphate (PLP)-dependent enzymes that convert _L_-lysine to cadaverine by a decarboxylation reaction^14^. Ironically, *C. glutamicum* is also known to lack LDC, which results in the accumulation of _L_-lysine^15^. Moreover, *Cg*2612 shows high amino acid similarity with LOG proteins, suggesting that *Cg*2612 is possibly an LOG protein. In this report, in order to elucidate the function of *Cg*2612, we determined its crystal structure. Based on biochemical studies and structural comparison with other LOGs, we propose that *Cg*2612 functions as an LOG. In addition, we identified key residues responsible for enzyme catalysis and substrate binding.

## Results

### Overall structure of *Cg*2612

To investigate the function of *Cg*2612, we determined its crystal structure at a 2.3 Å resolution (Table 1). The asymmetric unit contained four molecules and seems to contain two distinct dimers. Molecules I, II, III, and IV of *Cg*2612 contain 9-191, 3-195, 3-190, and 2-195 residues visible in the electron density map, respectively. The R.M.S.D. values between these four monomeric structures are under 0.4, indicating that four monomers have quite similar structures each other. Interestingly, among four monomers in the asymmetric unit, two monomers contain a phosphate ion at each active site. We found that Lys194 from one dimer interacts with a phosphate in the active site of the other dimer (Supplementary Fig. S1). We speculate that crystal packing in *I*222 space group caused tetrameric arrangement as an artifact (Supplementary Fig. S1). Size-exclusion chromatography analysis suggested that *Cg*2612 forms a dimer (Supplementary Fig. S2). We then performed small-angle X-ray scattering (SAXS) experiment to further confirm the dimeric conformation of *Cg*2612 in solution, and the result indicates that *Cg*2612 functions as a dimer as observed in other LOGs (Supplementary Fig. S3).

**Figure 1:**
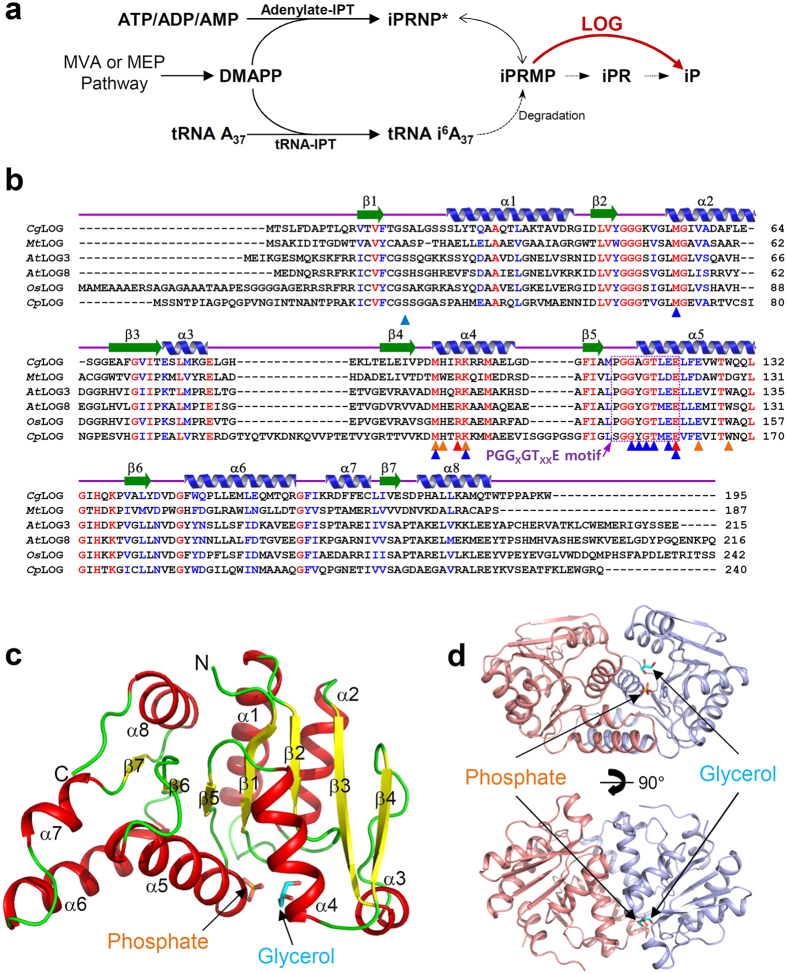
Overall structure of *Cg*LOG. (**a**) Biosynthetic pathway for cytokinin production. (**b**) Amino acid sequence alignment of LOGs. The secondary structure elements are drawn based on the structure of *Cg*LOG. Residues involved in catalysis, AMP binding, and prenyl-group binding are indicated by red, blue, and orange-colored triangles, respectively. The PGG_X_GT_XX_E motif is indicated with a purple-colored dotted rectangle. *Cg*LOG, *Mt*LOG, *At*LOG, *Os*LOG, and *Cp*LOG are abbreviations of LOGs from *Corynebacterium glutamicum*, *Mycobacterium tuberculosis*, *Arabidopsis thaliana*, *Oryza sativa*, and *Claviceps purpurea*, respectively. (**c**) The monomeric structure of *Cg*LOG presented as a cartoon diagram. The bound glycerol and phosphate molecules are shown as stick models. Secondary structure elements are labeled. (**d**) Dimeric structure of *Cg*LOG. The dimeric structure of *Cg*LOG is presented as a cartoon diagram. The bound glycerol and phosphate molecules are shown as in (**c**). The bottom figure is the top figure rotated horizontally by 90°.

The monomeric structure of *Cg*2612 shows an α/β fold belonging to a Rossmann fold (Fig. 1b,c). The central β-sheet which is formed by seven parallel β-strands is surrounded by eight α-helices (Fig. 1c). Dimerization of *Cg*2612 displays a compact domain folding. The dimerization interface is mainly composed of α5- and α6-helices, and the α4-helix partially aids in dimerization (Fig. 1d and Supplementary Fig. S4). PISA^16^ computed the buried interface area to be 1,563 Å (averaged with AB dimer and CD dimer) and the percentage of participating residues to be 24.5%. Dimerization of two polypeptides constitutes a pocket which serves as the active site, and the conserved “PGG_X_GT_XX_E” motif was found in the surface of the pocket, which will be described in detail later.

### *Cg*2612 has LOG function

**Figure 2:**
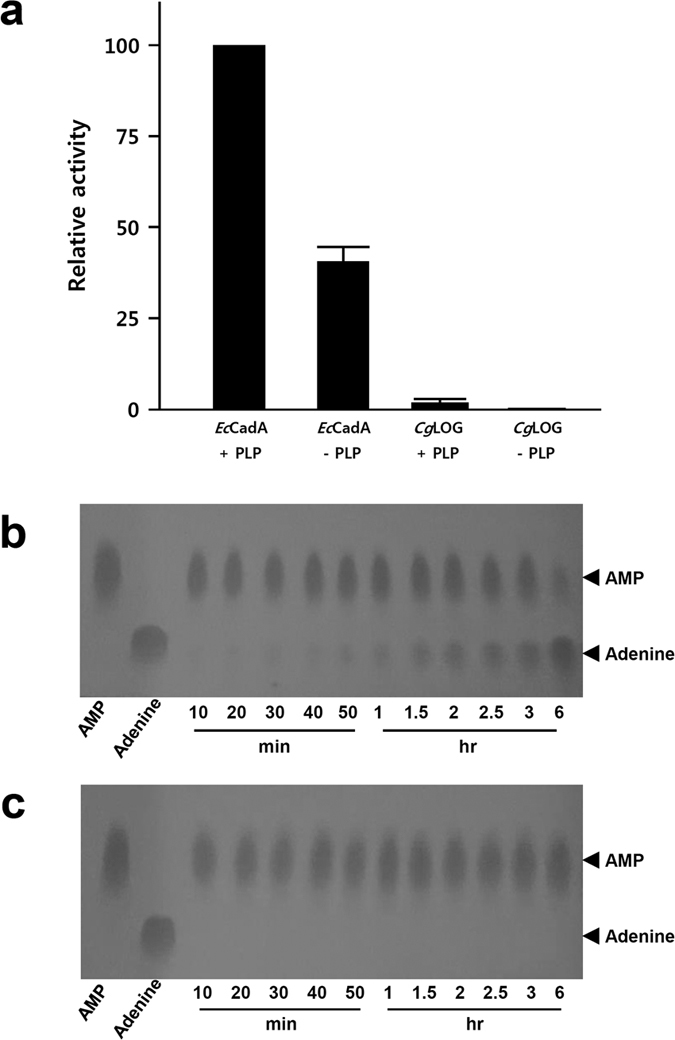
Phosphorobohydrolase activity of *Cg*LOG. (**a**) Lysine decarboxylase activity assay of *Ec*CadA and *Cg*LOG. The lysine decarboxylase activity of *Ec*CadA and *Cg*LOG are measured with and without PLP. All experiments are performed in triplicates. (**b,c**) Phosphorobohydrolase activity of *Ec*CadA (**b**) and *Cg*LOG (**c**). The phosphorobohydrolase activity was detected by spotting and running the reaction mixture on TLC. The AMP and adenine standard are indicated. The incubation time of the reaction is labeled on the bottom of the figure.

Structural comparison using the DALI server^17^ showed that the structure of *Cg*2612 is quite similar to LOG3 (*At*LOG3, PDB CODE 2A33, Z-score 29.4) and LOG8 (*At*LOG8, PDB CODE 1YDH, z-score 30.4) from *A. thaliana*. The comparison also showed that LOGs from *C. purpurea* (*Cp*LOG, PDB CODE 5AJT, Z-score 26.8) and *M. marinum* (*Mm*LOG, PDB CODE 3SBX, Z-score 27.7) are structural homologs of *Cg*2612. These structural homologs also shared amino acid identity higher than 33% with *Cg*2612. Because these structural homologs of *Cg*2612 have been identified as LOG proteins, high similarity in structure and amino acid sequence with these proteins suggests that *Cg*2612 functions as an LOG. To investigate the biochemical function of *Cg*2612, we performed lysine decarboxylase and phosphoribohydrolase activity assays on *Cg*2612, and compared the results with lysine decarboxylase from *E. coli* (*Ec*CadA). As expected, *Cg*2612 did not show any lysine decarboxylase activity, while *Ec*CadA showed strong activity (Fig. 2a). These results indicate that *Cg*2612 is not a lysine decarboxylase as inferred from previous studies on LOGs and high amino acid sequence identity of *Cg*2612 with LOGs. We then tested if *Cg*2612 has a phosphoribohydrolase activity. For this assay, we used adenosine monophosphate (AMP) as a substrate, because we could not obtain natural cytokinin precursors and it was previously reported that LOG has a phosphoribohydrolase activity against an AMP substrate^9^. Interestingly, we observed that *Cg*2612 has phosphoribohydrolase activity and this activity tends to increase upon reaction time (Fig. 2b). However, LOGs generally show higher phosphoribohydrolase activity against natural cytokinin precursors than AMP^5^,^9^, suggesting that *Cg*2612 might have much higher phosphoribohydrolase activity against natural cytokinin precursors than observed with AMP as a substrate. On the other hand, *Ec*CadA showed no phosphoribohydrolase activity with AMP as a substrate (Fig. 2c). These results confirm that *Cg*2612 belongs to the LOG family, and hereafter, we will represent *Cg*2612 as *Cg*LOG.

### Active site of *Cg*LOG

**Figure 3:**
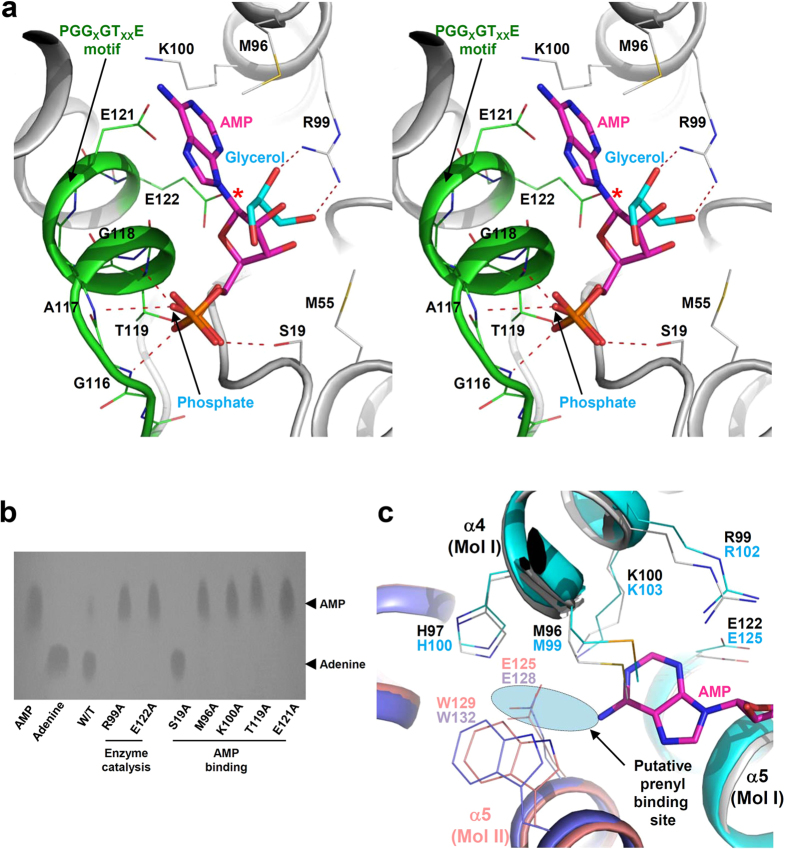
Active site of *Cg*LOG. (**a**) Stereo view of the *Cg*LOG AMP binding site. The *Cg*LOG structure is superposed with LOG from *M. marinum* (*Mm*LOG) in complex with AMP. The bound AMP molecule in *Mm*LOG is shown with the *Cg*LOG structure. Secondary structure elements are labeled. *Cg*LOG is shown as a cartoon diagram. Residues involved in AMP binding are shown as line models. The bound AMP molecule was prepared as in Fig. 1b. The bound glycerol and phosphate molecules are labeled and shown as stick models. The hydrogen bonds involved in stabilization of the glycerol and phosphate molecules are shown with red dotted lines. The PGG_X_GT_XX_E motif is depicted with a green color. The covalent bond (a bond between adenine-N^9^ and ribose-C^1^) hydrolyzed by the enzyme is indicated by a star symbol. (**b**) Site-detected mutagenesis experiments. The AMP and adenine standard are indicated. (**c**) Prenyl-group binding site. The *Cg*LOG structure is superposed with *At*LOG3. Two monomers of *Cg*LOG are distinguished with colors of grey and salmon and those of *At*LOG3 are with colors of cyan and light-blue. The bound AMP molecule is shown as a stick model in magenta. Two catalytic residues (R39 and E122 in *Cg*LOG) and residues involved in the constitution of the prenyl-group binding site are shown as line models. The putative prenyl-group binding site is labeled and shown with a cyan circle.

In order to elucidate an active site and a substrate binding mode of *Cg*LOG, we tried to determine the structure in complex with AMP or cytokinin. However, neither co-crystallization nor soaking of AMP or cytokinin into the *Cg*LOG crystal was successful. We then superposed our structure with *Mm*LOG in complex with AMP^18^. The active site of *Cg*LOG is located near the “PGG_X_GT_XX_E” motif. The phosphate moiety was hydrogen bonded with main chains of Gly116, Ala117, and Gly118, and side chains of Thr119 and Ser19 (Fig. 3a). The ribose moiety is mainly stabilized by hydrogen bond interactions between Arg99 and two hydroxyl groups of the ribose moiety (Fig. 3a). To stabilize the adenine ring, a mixture of hydrophobic and hydrophilic residues, Met96, Lys100, and Glu121, form an adenine binding site (Fig. 3a). Two proposed catalytic residues, Arg99 and Glu122, are located in the vicinity of the bond to be hydrolyzed; a covalent bond between adenine-N^9^ and ribose-C^1^ (Fig. 3a). Among the residues involved in AMP binding and enzyme catalysis, Gly116, Ala117, Gly118, Thr119, Glu121, and Glu122 are located in the “PGG_X_GT_XX_E” motif, indicating that the motif serves as a nucleotide binding site as suggested by other LOG structures. In our current structure, one phosphate and one glycerol molecule are bound at the AMP binding site and these molecules mimic the stabilization of the phosphate moiety and the ribose ring, respectively (Fig. 3a and Supplementary Fig. S5). In order to confirm the involvement of these residues in AMP binding and enzyme catalysis, we performed site-directed mutagenesis experiments. As expected, substituting these crucial residues with alanines resulted in almost complete loss of phosphoribohydrolase activity (Fig. 3b). One exception is S19A mutant which shows higher activity than the wild-type. Most of the residues that involved in AMP stabilization are conserved in all LOGs, and the active site conformation observed in *Cg*LOG further supports the classification of this protein as a LOG family enzyme (Fig. 1b).

Stabilization of the prenyl-group, the N^6^-modifying moiety of cytokinin precursors, still remains unclear due to the absence of a LOG structure in complex with a natural substrate or cytokinin. However, the binding site of the N^6^-prenyl group could be inferred from configuration of the adenine moiety and positioning of N^6^ atom of AMP bound in *Mm*LOG. Superposition of the *Cg*LOG structure with *Mm*LOG in complex with AMP also leads us to speculate the prenyl-group binding site of *Cg*LOG. In *Cg*LOG, Met96, His97, Lys100, Glu125, and Trp129 seem to form a prenyl-group binding site (Fig. 3c). The prenyl-group binding locates at the dimer interface and especially Glu125 and Trp129 residues are provided from a neighboring molecule (Fig. 3c). Importantly, these residues are identical to those found in *At*LOG3, *Os*LOG, and *Cp*LOG (Figs 1b and 3c). Based on these observations, we propose that *Cg*LOG utilizes cytokinin precursors as substrates that are similarly used by other LOGs from plants or plant-interacting fungus.

### Structural comparison of *Cg*LOG with other LOGs

**Figure 4:**
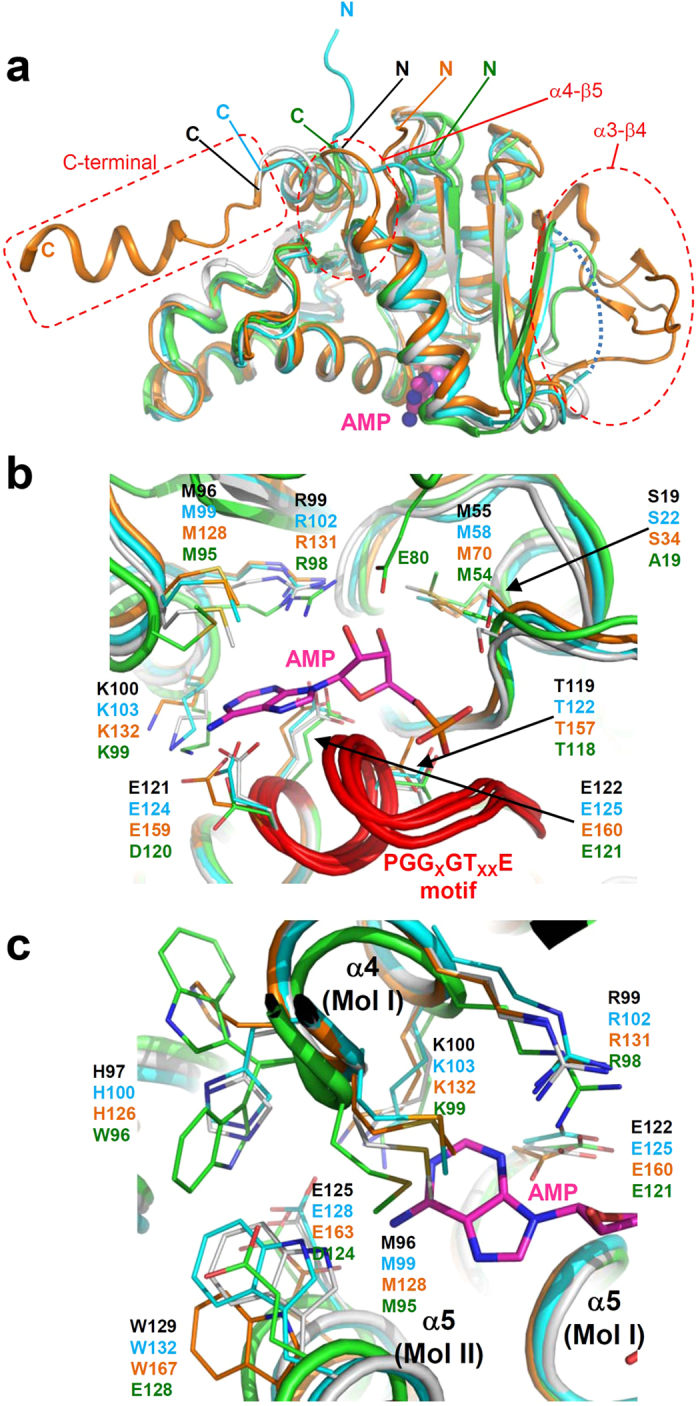
Structural comparison of *Cg*LOG with other LOGs. (**a**) Comparison of overall structure. Monomeric structures of *Cg*LOG, *At*LOG, *Cp*LOG, and *Mm*LOG are superposed and presented as cartoon diagrams in grey, cyan, orange, and green, respectively. The AMP molecule bound in the *Mm*LOG structure is shown in magenta as a stick model. The N-terminus and the C-terminus of LOGs are indicated. Structural differences found in *Cp*LOG are indicated with red dotted circles and labeled. The distorted connecting loop of α3-β4 in *At*LOG3 is shown with a cyan dotted line. (**b**) Structural comparison in AMP binding sites. Monomeric structures of *Cg*LOG, *At*LOG, *Cp*LOG, and *Mm*LOG are superposed and presented with color scheme as in (**a**). Residues of four LOGs involved in AMP binding are shown as line models and labeled with the same color scheme as in (**a**). The “PGG_X_GT_XX_E” motifs of LOGs are distinguished with a red color and labeled. (**c**) Structural comparison of prenyl-group binding sites. Dimeric structures of *Cg*LOG, *At*LOG, *Cp*LOG, and *Mm*LOG are superposed and presented with color scheme as in (**a**). Residues of four LOGs involved in the constitution of the putative prenyl-group binding site are shown as line models and labeled with the same color scheme as in (**a**).

To compare *Cg*LOG with other LOGs, we superposed the *Cg*LOG structure with other LOG proteins such as *At*LOG3, *Cp*LOG, and *Mm*LOG. Although the overall folds of all four LOG structures are quite similar to each other, *Cp*LOG exhibited somewhat unique structural features. Compared with the three other LOGs, *Cp*LOG has an extra helix in the C-terminal region and contains extended connecting loops of α3-β4 and α4-β5 (Fig. 4a). Notably, the extended connecting loop of α3-β4 is located near the AMP binding site (Fig. 4a). In *At*LOG3 this region is distorted, in *Cg*LOG this region showed high a b-factor. However, in *Mm*LOG, this region contains the Glu80 residue that forms direct a hydrogen bond with the hydroxyl group of the ribose ring (Fig. 4b). These observations indicate that this region is quite diverse in various LOGs and stabilization of the ribose ring seems to occur somewhat differently in each protein. Except for structural differences in this region, LOGs have similar AMP binding modes (Fig. 4b). The conserved residues in “PGG_X_GT_XX_E” motifs along with other conserved residues contribute to AMP stabilization (Figs 1b and 4b and Supplementary Fig. S6). One exception is found in *Mm*LOG; Ala19 and Asp120 are involved in AMP stabilization while serine and glutamate residues are located at the corresponding positions in other LOGs (Fig. 4b). Moreover, all four LOGs have two catalytic residues, and Arg99 and Glu122 in *Cg*LOG are located at the same positions, indicating that these LOGs catalyze this reaction via the same catalytic mechanism. The comparison of the prenyl-group binding sites provides insights into LOG substrate specificity. As observed in *Cg*LOG, *At*LOG3 and *Cp*LOG contain the residues Met96, His97, Lys100, Glu125, and Trp129 at the prenyl-group binding site (Fig. 4c). However, *Mm*LOG has a glaring discrepancy in the prenyl-group binding site compared with *Cg*LOG, *At*LOG3, and *Cp*LOG. At the prenyl-group binding site in *Mm*LOG, Asp124, Glu128, and Trp96 residues are located at the positions corresponding to glutamate, tryptophan, and histidine residues, respectively, in the other three LOGs (Fig. 4c). These comparisons suggest that *Cg*LOG might produce cytokinins similar to those produced by LOGs from plants and plant-interacting fungi. However, *Mm*LOG might produce different types of cytokinins than *Cg*LOG, *At*LOG3 and *Cp*LOG, which drives us to speculate that mammalian-interacting bacteria like those in the *Mycobacterium* genus seem to utilize different types of cytokinins.

## Discussion

Our structural and biochemical studies on *Cg*2612, a protein previously annotated as a LDC, imply that this protein functions as LOG. Because LOGs are enzymes that catalyze the production of cytokinins, here we can raise the question if *C. glutamicum* truly synthesizes cytokinins. As we mentioned above, the first reaction for cytokinin production is the prenylation of ATP/ADP/AMP or tRNA by IPTs, and these enzymes can be divided into two categories depending on whether they utilize nucleotides or tRNA as a substrate. Adenylate-IPT is usually found in higher plants or phytopathogens (known as *Fas4*) as a main regulator for cytokinin levels, while tRNA-IPT (*MiaA*) is ubiquitous to improve the efficiency and fidelity of the codon-anticodon interaction during translation^19^. It was reported that *M. tuberculosis* H37Rv produces cytokinins such as iP and tZ^9^. Interestingly, genome analysis of *M. tuberculosis* H37Rv revealed that this strain contains a gene coding for tRNA-IPT (*Rv2727c*, *LH57*14920, *MiaA*), but not for adenylate-IPT. This analysis intimated that the tRNA-mediated cytokinin activation is a major pathway for cytokinin production in bacteria, although it was considered to be a minor pathway in *Arabidopsis*^20^. Like *M. tuberculosis*, the genome of *C. glutamicum* ATCC 13032 only contains a gene coding for tRNA-IPT (*Cg2130, MiaA*). In addition, *Cg*2130 exhibits 56.9% amino acid sequence identity with *Rv*2727c. These results indicate that *C. glutamicum* seems to produce cytokinins by a tRNA-mediated activation mechanism similar to the mechanism in *M. tuberculosis*. Thus, studies on cytokinin detection and biosynthesis in *C. glutamicum* are needed.

Some soil bacteria communicate with plants via cytokinins. For instance, *Rhodococcus fascians* produces cytokinins to infect to a wide range of plants and causes diseases in plant hosts such as a leafy gall syndrome^21^. The virulence of *C. glutamicum* in plant species has not yet been reported. However, because *C. glutamicum* is a soil-dwelling bacterium in nature, investigating *C. glutamicum*-plant communication through cytokinins is of interest for agricultural applications. Furthermore, recent studies on cytokinin production in *M. tuberculosis*, a mammalian pathogen, suggest that cytokinin production by microbes is not limited to communication with plants, but rather can be applied to wide cross-kingdom communications^9^,^22^.

## Methods

### Protein preparations

The gene coding for LOG from *Corynebacterium glutamicum* ATCC 13032 (*Cg*LOG) was amplified from chromosome of *C. glutamicum* by polymerase chain reaction (PCR) with primers: forward, 5-GCGC**CATATG**ACTTCGCTTTTCGACGCCCC-3, and reverse, 5-GCGC**CTCGAG**CCATTTTGGTGCTGGTGGAGTCC-3. The PCR product was then subcloned into pET30a (Novagen) with 6xHis at the C-terminus. The resulting expression vectors pET30a: *CgLOG* was transformed into *E. coli* BL21 (DE3) strain and was grown on LB medium containing 100 mg l^−1^ kanamycin at 37 °C to OD600 of 0.6. After induction with 1.0 mM 1-thio-β-D-galactopyranoside (IPTG) for a further 20 h at 18 °C, the culture was harvested by centrifugation at 5,000 × g for 15 min at 4 °C. The cell pellet was resuspended in ice-cold buffer A (40 mM Tris–HCl, pH 8.0) and disrupted by ultrasonication. The cell debris was removed by centrifugation at 11,000 × g for 1 h, and lysate was bound to Ni-NTA agarose column (Qiagen). After washing with buffer A containing 20 mM imidazole, the bound proteins were eluted with 300 mM imidazole in buffer A. Further purification was carried out by applying the HiTrap Q ion exchange chromatography and size exclusion chromatography. The purified proteins were concentrated to 30 mg ml^−1^ in 40 mM Tris–HCl, pH 8.0, and stored at −80 °C for crystallization trials. Site-directed mutagenesis experiments were performed using the QuikChange site-directed mutagenesis kit (Stratagene). The production and purification of the *Cg*LOG mutants were carried out by the same procedures as described for the wild-type protein. CadA from *E. coli* (*Ec*CadA) was prepared by the procedure similar to *Cg*LOG.

### Crystallization, data collection and structure determination

Crystallization of the purified proteins were initially performed by the hanging-drop vapor-diffusion method at 20 °C using commercially available sparse-matrix screens from Hampton Research and Emerald BioSystems. Each experiment consisted of mixing 1.0 μl protein solution with 1.0 μl reservoir solution and then equilibrating it against 0.5 ml of the reservoir solution. The *Cg*LOG crystals were observed from several crystallization screening conditions. After several optimization steps using the hanging-drop vapor-diffusion method, the best-quality crystals appeared in 2 day using a reservoir solution consisting of 0.2 M _DL_-malic acid, pH 7.0 and 24% PEG 3350 and reached maximal dimensions of approximately 0.3 × 0.3 × 0.1 mm. For the cryo-protection the crystals, glycerol of 30% glycerol in reservoir solution was used. Data were collected at the 7A beamline at the Pohang Accelerator Laboratory using a QUANTUM 270 CCD detector (San Diego, CA, USA) at the wavelength of 0.97934 Å. The CgLOG crystal diffracted to resolution of 2.3 Å. The data was then indexed, integrated, and scaled using the HKL2000 program^23^. Crystals of *Cg*LOG belonged to the I-centered orthorhombic space group *I*222 with unit cell dimensions of *a* = 113.51 Å *b* = 130.50 Å *c* = 140.51 Å. With four *Cg*LOG molecules per asymmetric unit, the crystal volume per unit of protein mass was approximately 3.10 Å^3^ Da^−1^, which corresponds to a solvent content of approximately 60.31%^24^. The structure of *Cg*LOG was solved by molecular replacement method using *MOLREP*^25^ with LOG from *A. thaliana* (*At*LOG, PDB CODE 2A33) as a search model. The model building was performed using the program *WinCoot*^26^ and the refinement was performed with *REFMAC5*^27^. The data statistics are summarized in Table 1. The refined models of *Cg*LOG was deposited in the Protein Data Bank (PDB CODE 5ITS).

### Solution SAXS measurements

Small-angle X-ray scattering (SAXS) measurements were carried out using the 4C SAXS II beamline of the Pohang Accelerator Laboratory (Pohang, Korea). A sample-to-detector distance (SDD) of 4.00 m and 1.00 m for SAXS were used. The magnitude of scattering vector, *q* = (4*π/λ*) sin *θ*, was 0.1 nm^−1^ < *q* < 6.50 nm^−1^, where 2*θ* is the scattering angle and *λ* is the wavelength of the X-ray beam source. All scattering measurements were carried out at 4 °C by using a FP50-HL refrigerated circulator (JULABO, Germany). The SAXS data were collected in six successive frames of 0.1 min each to monitor radiation damage. Measurements of *Cg*LOG were carried out over a small concentration range 0.5 ~ 4.5 mg/ml. Each 2D SAXS pattern was radial averaged from the beam center and normalized to the transmitted X-ray beam intensity, which was monitored with a scintillation counter placed behind the sample. The R_g,G_ (radius of gyration) values were estimated from the scattering data using Guinier analysis^28^. The molecular mass (MM) was calculated from the scattering curve based on the Q_R_ method^29^. The pair distance distribution p(r) function was obtained through the indirect Fourier transform method using the program GNOM^30^.

### Lysine decarboxylase activity assay

The activity of LDC was determined by measuring residual concentration of _L_-lysine using lysine oxidase and peroxidase. After LDC reaction, lysine oxidase converts remaining lysine into 6-amino-2-oxohexanoate, NH_3_, and H_2_O_2_ and then the hydrogen peroxide is reduced by peroxidase with 2,2′-azino-bis(3-ethylbenzothiazoline-6-sulphonic acid) (ABTS). The oxidized ABTS is detected by spectrophotometric method in absorbance at 412 nm. The assay was performed at 30 °C in a total volume 200 μl, containing 100 mM potassium phosphate, pH 6.0, 0.1 M _L_-lysine, 0.2 mM pyridoxal-5-phosphate, and 25 μg of purified enzymes. The reaction was stopped by heating the reaction mixture at 100 °C for 5 min. After centrifugation at 13,500 × *g* for 1 min, 2X reaction solution that contains 0.1 unit ml^−1^ lysine oxidase and 1 unit ml^−1^ peroxidase in potassium phosphate buffer is added to the reaction mixture.

### Phosphoribohydrolase activity assay

The phosphoribohydrolase activity was determined by detecting adenine ring compounds separated by thin layer chromatography (TLC) method. Enzyme reactions were carried out in the mixture of 20 mM AMP, 36 mM Tris-HCl, pH 8.0, and 23 μM purified enzymes at 30 °C and then the reactions were stopped by heating the mixture at 95 °C for 1.5 min. The reaction mixtures were then dotted on PEI-cellulose-F plastic TLC sheet (Merck Millipore). The mobile phase was 1 M sodium chloride. After development in the TLC chamber, the sheet was dried completely. Adenine ring-including compounds were detected by UV lamp (290 nm).

### Size-exclusion chromatographic analysis

To investigate the oligomerization of *Cg*LOG, analytical size-exclusion chromatography was performed using a Superdex 200 10/300 column (GE Healthcare) at NaCl concentrations of 150 mM. 300 μL of protein samples with concentration of 3 mg/ml were analyzed. The molecular weights of the eluted samples were calculated based on the calibration curve of standard samples.

## Additional Information

**How to cite this article**: Seo, H. *et al*. Structural basis for cytokinin production by LOG from *Corynebacterium glutamicum*. *Sci. Rep*. **6**, 31390; doi: 10.1038/srep31390 (2016).

## Supplementary Material

Supplementary Information

## Figures and Tables

**Table 1:** Data collection and structural refinement statistics.

	*Cg*LOG
**Data collection**	
Space group	*I222*
Cell dimensions	
*a*, *b*, *c* (Å)	113.5, 130.5, 140.5
α, β, γ (°)	90.00, 90.00, 90.00
Resolution (Å)	50.00–2.30 (2.34–2.30)
*R*_sym_ or *R*_merge_	10.2 (31.9)
*I*/σ*I*	17.5 (3.5)
CC1/2	0.989 (0.63)
Completeness (%)	95.2 (87)
Redundancy	6.8 (3.2)
**Refinement**	
Resolution (Å)	50.00–2.30
No. reflections	41951
*R*_work_/*R*_free_	18.3/23.0
No. atoms	6149
Protein	5899
Glycerol/PO_4_^3−^	36/10
Water	204
*B*-factors	29.69
Protein	29.76
Glycerol/PO_4_^3−^	38.04/20.20
Water	30.13
R.m.s. deviations	
Bond lengths (Å)	0.015
Bond angles (°)	1.704

^a^The numbers in parentheses are statistics from the highest resolution shell.

^b^*R*_sym_ = Σ |*I*_obs_ − *I*_avg_|/*I*_obs_, where *I*_obs_ is the observed intensity of individual reflection and *I*_avg_ is average over symmetry equivalents.

^c^*R*_work_ = Σ ||*F*_o_| − |*F*_c_||/Σ |*F*_o_|, where |*F*_o_| and |*F*_c_| are the observed and calculated structure factor amplitudes, respectively. *R*_free_ was calculated with 5% of the data.

## References

[b1] Skoog F. & Armstrong D. J. Cytokinins. Annual review of plant physiology 21, 359–384 (1970).

[b2] Sakakibara H. Cytokinins: activity, biosynthesis, and translocation. Annu Rev Plant Biol 57, 431–49 (2006). 16669769 10.1146/annurev.arplant.57.032905.105231

[b3] Mok D. W. & Mok M. C. Cytokinin Metabolism and Action. Annu Rev Plant Physiol Plant Mol Biol 52, 89–118 (2001). 11337393 10.1146/annurev.arplant.52.1.89

[b4] Kakimoto T. Identification of plant cytokinin biosynthetic enzymes as dimethylallyl diphosphate: ATP/ADP isopentenyltransferases. Plant and Cell Physiology 42, 677–685 (2001). 11479373 10.1093/pcp/pce112

[b5] Hinsch J. . De novo biosynthesis of cytokinins in the biotrophic fungus Claviceps purpurea. Environmental microbiology (2015). 10.1111/1462-2920.12838 25753486

[b6] Kurakawa T. . Direct control of shoot meristem activity by a cytokinin-activating enzyme. Nature 445, 652–5 (2007). 17287810 10.1038/nature05504

[b7] Kukimoto-Niino M. . Crystal structures of possible lysine decarboxylases from Thermus thermophilus HB8. Protein Sci 13, 3038–42 (2004). 15459330 10.1110/ps.041012404 PMC2286578

[b8] Kuroha T. . Functional analyses of LONELY GUY cytokinin-activating enzymes reveal the importance of the direct activation pathway in Arabidopsis. Plant Cell 21, 3152–69 (2009). 19837870 10.1105/tpc.109.068676 PMC2782294

[b9] Samanovic M. I. . Proteasomal control of cytokinin synthesis protects Mycobacterium tuberculosis against nitric oxide. Mol Cell 57, 984–94 (2015). 25728768 10.1016/j.molcel.2015.01.024 PMC4369403

[b10] Jeon W. B. . X-ray crystal structures of the conserved hypothetical proteins from Arabidopsis thaliana gene loci At5g11950 and AT2g37210. Proteins 65, 1051–4 (2006). 17048257 10.1002/prot.21166

[b11] Dzurova L. . The three-dimensional structure of “Lonely Guy” from Claviceps purpurea provides insights into the phosphoribohydrolase function of Rossmann fold-containing lysine decarboxylase-like proteins. Proteins 83, 1539–46 (2015). 26010010 10.1002/prot.24835

[b12] Vertes A. A., Inui M. & Yukawa H. Manipulating corynebacteria, from individual genes to chromosomes. Appl Environ Microbiol 71, 7633–42 (2005). 16332735 10.1128/AEM.71.12.7633-7642.2005 PMC1317429

[b13] Hayashi M. . Transcriptome analysis reveals global expression changes in an industrial L-lysine producer of Corynebacterium glutamicum. Bioscience, biotechnology, and biochemistry 70, 546–550 (2006). 10.1271/bbb.70.546 16495679

[b14] Purich D. L. & Allison R. D. The enzyme reference: a comprehensive guidebook to enzyme nomenclature, reactions, and methods (Academic press, 2003).

[b15] Kalinowski J. . The complete Corynebacterium glutamicum ATCC 13032 genome sequence and its impact on the production of L-aspartate-derived amino acids and vitamins. J Biotechnol 104, 5–25 (2003). 12948626 10.1016/s0168-1656(03)00154-8

[b16] Krissinel E. & Henrick K. Inference of macromolecular assemblies from crystalline state. Journal of molecular biology 372, 774–797 (2007). 17681537 10.1016/j.jmb.2007.05.022

[b17] Holm L. & Sander C. Touring protein fold space with Dali/FSSP. Nucleic Acids Res 26, 316–9 (1998). 9399863 10.1093/nar/26.1.316 PMC147193

[b18] Baugh L. . Increasing the structural coverage of tuberculosis drug targets. Tuberculosis (Edinb) 95, 142–8 (2015). 25613812 10.1016/j.tube.2014.12.003 PMC4361283

[b19] Persson B., Esberg B., Olafsson O. & Björk G. Synthesis and function of isopentenyl adenosine derivatives in tRNA. Biochimie 76, 1152–1160 (1994). 7748950 10.1016/0300-9084(94)90044-2

[b20] Miyawaki K. . Roles of Arabidopsis ATP/ADP isopentenyltransferases and tRNA isopentenyltransferases in cytokinin biosynthesis. Proceedings of the National Academy of Sciences 103, 16598–16603 (2006). 10.1073/pnas.0603522103 PMC1637627 17062755

[b21] Stes E., Vandeputte O. M., El Jaziri M., Holsters M. & Vereecke D. A successful bacterial coup d’etat: how Rhodococcus fascians redirects plant development. Annu Rev Phytopathol 49, 69–86 (2011). 21495844 10.1146/annurev-phyto-072910-095217

[b22] Naseem M., Sarukhanyan E. & Dandekar T. LONELY-GUY Knocks Every Door: Crosskingdom Microbial Pathogenesis. Trends Plant Sci (2015). 10.1016/j.tplants.2015.10.017 26777904

[b23] Otwinowski Z. & Minor W. Processing of X-ray diffraction data collected in oscillation mode. Methods Enzymol 276, 307–326 (1997). 10.1016/S0076-6879(97)76066-X 27754618

[b24] Matthews B. W. Solvent content of protein crystals. Journal of molecular biology 33, 491–497 (1968). 5700707 10.1016/0022-2836(68)90205-2

[b25] Vagin A. & Teplyakov A. Molecular replacement with MOLREP. Acta Crystallographica Section D: Biological Crystallography 66, 22–25 (2009). 20057045 10.1107/S0907444909042589

[b26] Emsley P. & Cowtan K. Coot: model-building tools for molecular graphics. Acta Crystallographica Section D: Biological Crystallography 60, 2126–2132 (2004). 15572765 10.1107/S0907444904019158

[b27] Murshudov G. N., Vagin A. A. & Dodson E. J. Refinement of macromolecular structures by the maximum-likelihood method. Acta Crystallographica Section D: Biological Crystallography 53, 240–255 (1997). 15299926 10.1107/S0907444996012255

[b28] Glatter O. & Kratky O. Small angle X-ray scattering (Academic press, 1982). 10.1016/0076-6879(79)61013-3 481226

[b29] Rambo R. P. & Tainer J. A. Accurate assessment of mass, models and resolution by small-angle scattering. Nature 496, 477–481 (2013). 23619693 10.1038/nature12070 PMC3714217

[b30] Semenyuk A. & Svergun D. GNOM-a program package for small-angle scattering data processing. Journal of Applied Crystallography 24, 537–540 (1991). 10.1107/S0021889812007662 PMC4233345 25484842

