# Supplementary material for: Structural basis for cytokinin production by LOG from Corynebacterium glutamicum
Source: Sci Rep. 2016 Aug 10;6:31390. doi: 10.1038/srep31390 (PMC4979012; doi:10.1038/srep31390)
Supplement: Supplementary Information [file srep31390-s1.pdf]

Structural basis for cytokinin production by LOG from *Corynebacterium glutamicum*

Hogyun Seo, Sangwoo Kim, Hye-Young Sagong, Hyeoncheol Francis Son, Kyeong Sik Jin, Il-Kwon Kim and Kyung-Jin Kim

Supplementary Information

This Supplementary Information contains 6 Supplementary Figures

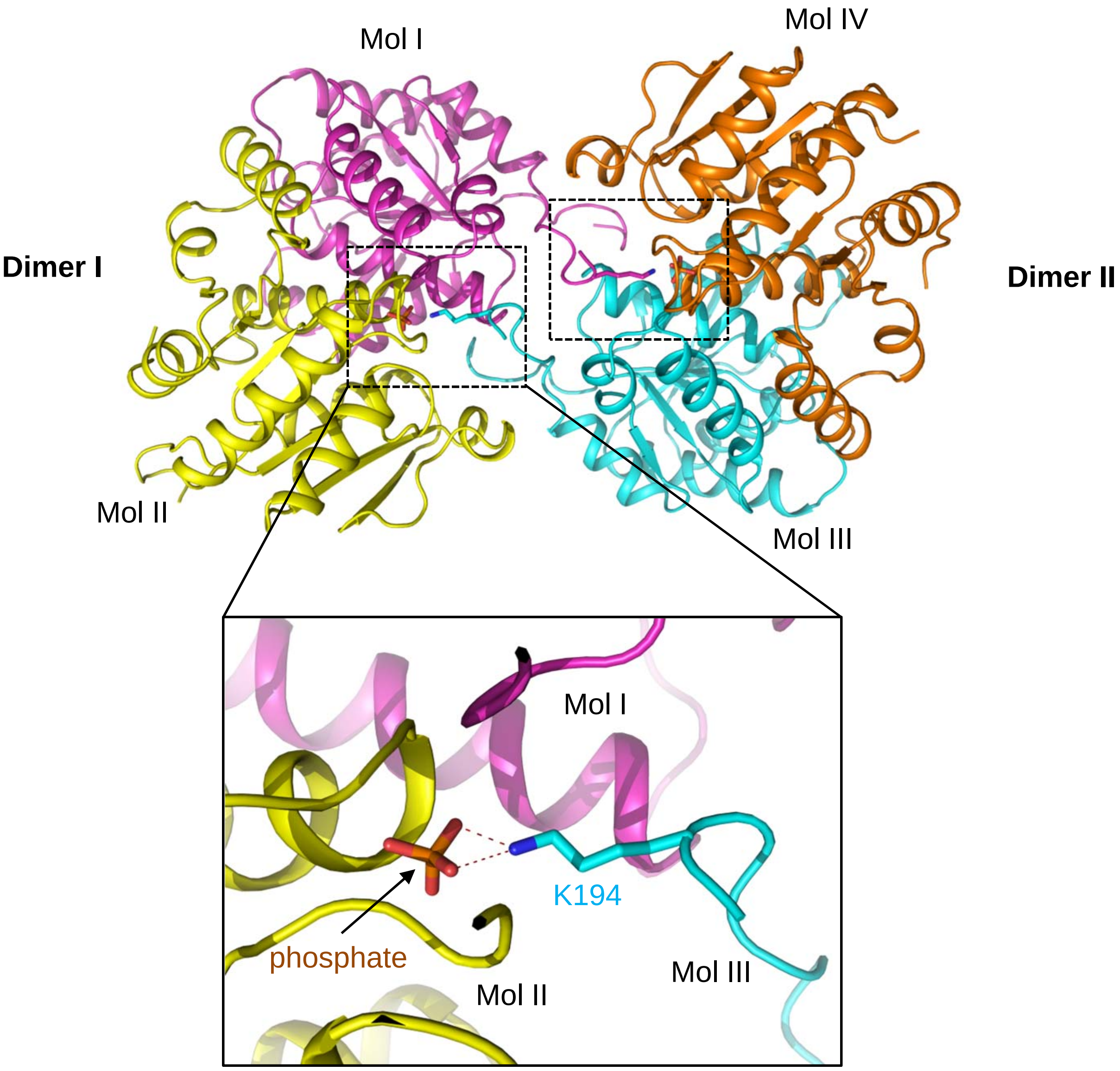

Supplementary Figure S1. Tetrameric structure of *Cg*LOG in I222 crystal packing. Tetrameric structure of *Cg*LOG in I222 crystal packing is drawn with a cartoon diagram, and each polypeptides are distinguished with different colors (top). Close-up view of interaction between two dimers (bottom). K194 from Mol III and a phosphate molecule bound in Mol II are shown as stick models with cyan and orange colors, respectively. The hydrogen bonds between K194 and the phosphate molecule are shown as dotted-lines.

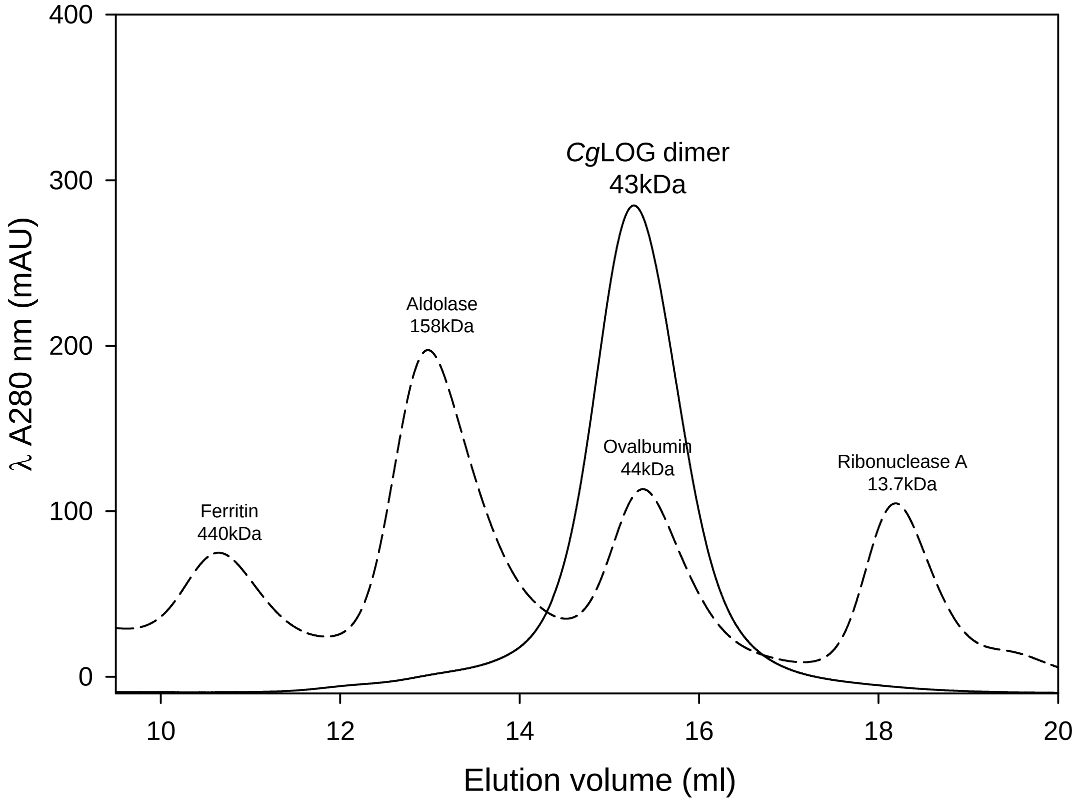

Supplementary Figure S2. Size-exclusion chromatographic analysis of *Cg*LOG. A elution pick corresponding to a dimeric state of *Cg*LOG in Size-exclusion chromatographic analysis. For precise analysis of the molecular weight, standard samples of ferritin (440kDa), aldolase (158kDa), ovalbumin (44kDa), and ribonuclease A (13.7kDa) are used for calibration and labelled.

a

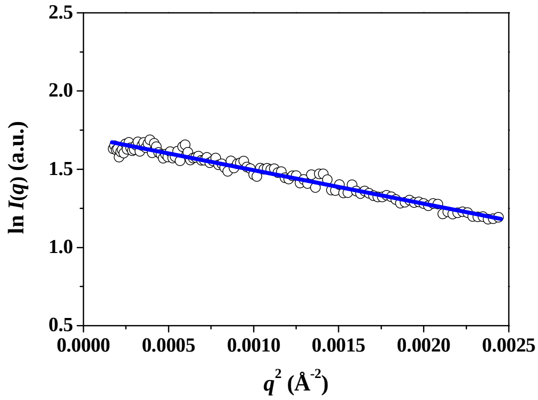

b

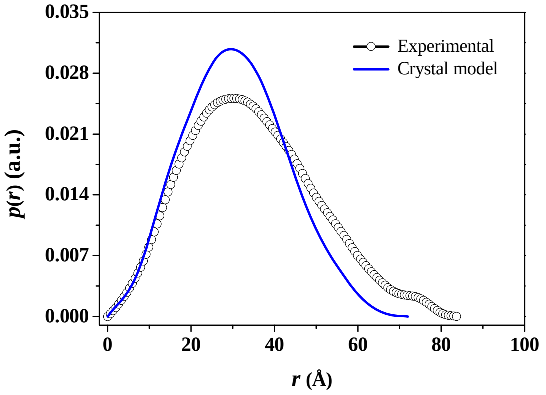

c

Sample	$R_{g,G}$ [a] (Å)	$R_{g,p(r)}$ [b] (Å)	D_{max} [c] (Å)	$MM_{calculated}$ [d] (kDa)	MM_{SAXS} [e] (kDa)	Conformation
Crystal	23.67 ± 0.01	23.45 ± 0.01	72	43	-	dimer
Experimental	26.08 ± 1.91	26.76 ± 0.46	84	43	48	dimer

[a] $R_{g,G}$ (radius of gyration) was obtained from the scattering data by the Guinier analysis.
[b] $R_{g,p(r)}$ (radius of gyration) was obtained from the p(r) function by the program GNOM.
[c] D_{max} (maximum dimension) was obtained from the p(r) function by the program GNOM.
[d] $MM_{calculated}$ (molecular mass) was obtained from the amino acid sequence of protein.
[e] MM_{SAXS} (molecular mass) was estimated from a BSA standard protein and the scattering curve based on the Q_R method.

Supplementary Figure S3. SAXS analysis of *Cg*LOG in aqueous solution. (a) Guinier plot of the X-ray scattering profiles of *Cg*LOG protein. (b) The pair distance distribution *p*(*r*) functions for *Cg*LOG protein in solution, based on an analysis of the experimental SAXS data using the program GNOM. (c) Structural parameters obtained from the SAXS data of *Cg*LOG in solution.

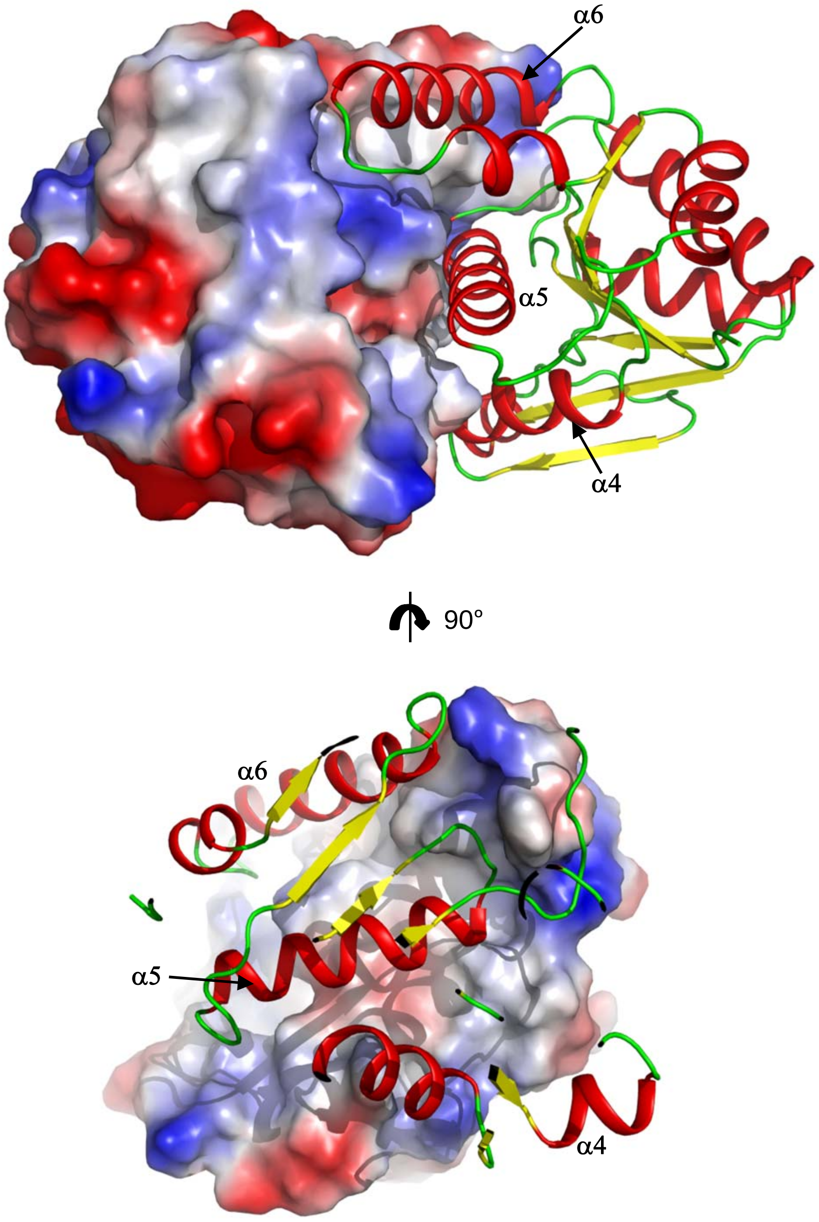

Supplementary Figure S4. Dimerization interface of *Cg*LOG. One monomer is shown as an electrostatic potential surface model and the other is as a cartoon diagram. The bottom figure is 90 degree rotation of the top figure in vertical direction. Three helices involved in the dimerization are labeled.

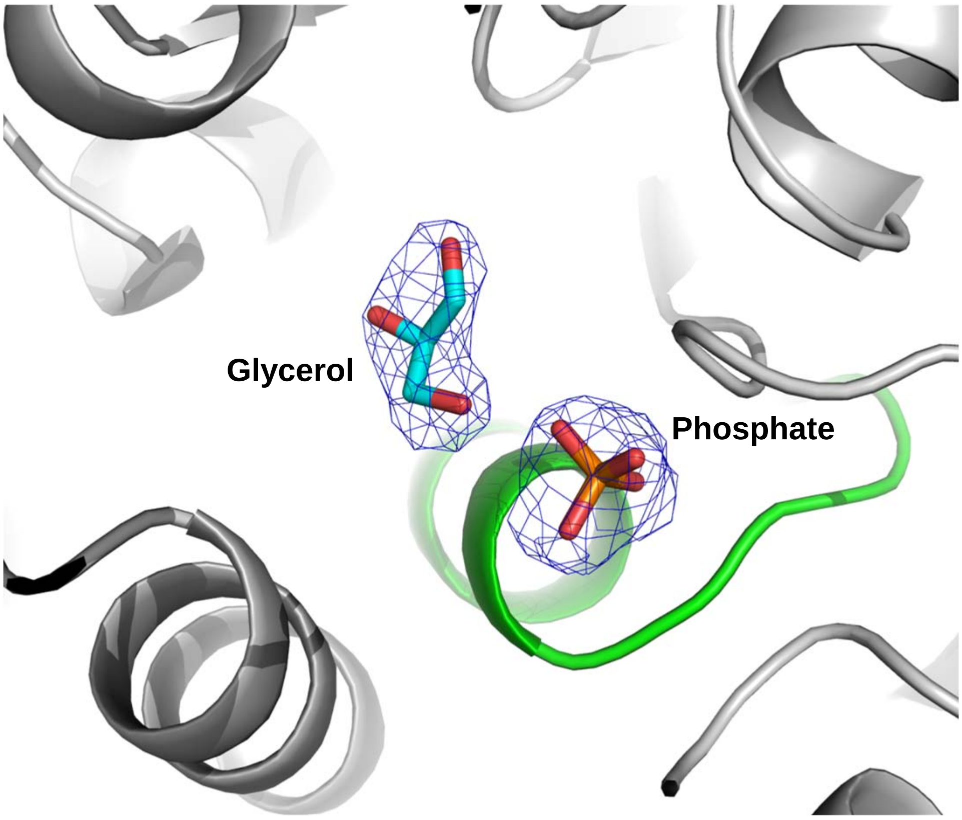

Supplementary Figure S5. Omit map of bound phosphate and glycerol molecules. The *Cg*LOG structure is shown as a cartoon diagram. The "PGG$_X$GT$_{XX}$E" motif located at the active site is distinguished with a green color. The omit maps of the bound phosphate and glycerol molecules are shown with a blue-colored mesh, and contoured at 4 σ.

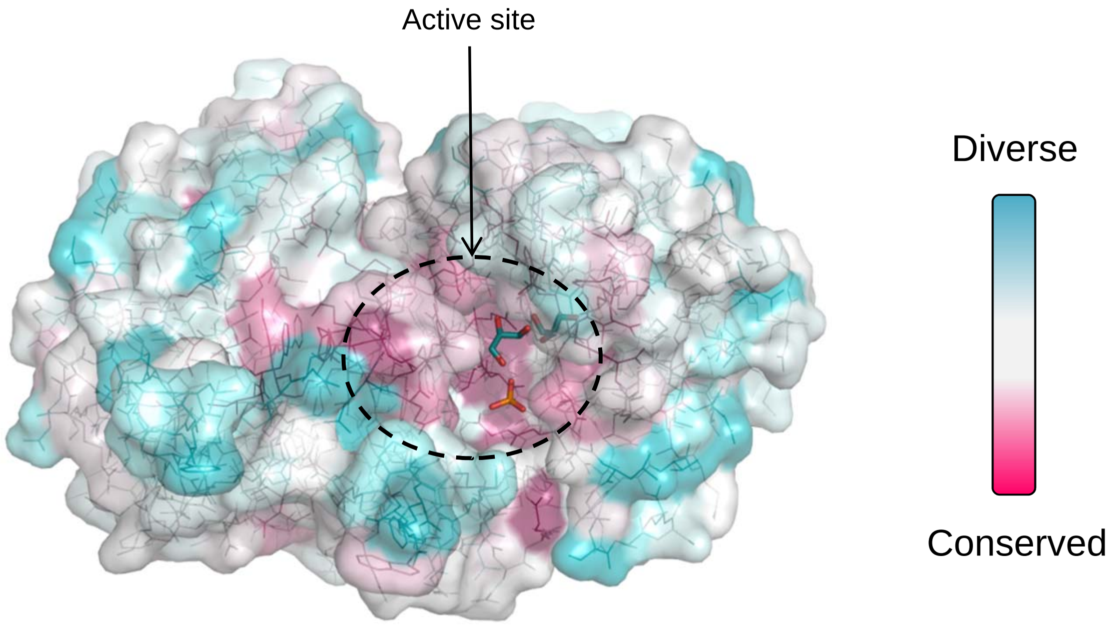

Supplementary Figure S6. Amino acid conservation in LOGs. The *Cg*LOG structure is shown as surface diagram with lines. Level of amino acid conservation is shown with different colors. The active site is indicated with a dotted-circle.
